# Proteome Analysis Revealed Changes in Protein Expression Patterns Caused by Mutations in *Ehrlichia chaffeensis*

**DOI:** 10.3389/fcimb.2019.00058

**Published:** 2019-03-18

**Authors:** Chandramouli Kondethimmanahalli, Huitao Liu, Roman R. Ganta

**Affiliations:** Department of Diagnostic Medicine/Pathobiology, Center of Excellence for Vector-Borne Diseases, College of Veterinary Medicine, Kansas State University, Manhattan, KS, United States

**Keywords:** *Ehrlichia chaffeensis*, intracellular bacteria, iTRAQ, quantitative proteomics, mutagenesis, 2-D gel electrophoresis

## Abstract

The tick-borne rickettsial pathogen, *Ehrlichia chaffeensis*, causes monocytic ehrlichiosis in people and other vertebrate hosts. Mutational analysis in *E. chaffeensis* genome aids in better understanding of its infection and persistence in host cells and in the development of attenuated vaccines. Our recent RNA deep sequencing study revealed that three genomic mutations caused global changes in the gene expression patterns, which in turn affect the ability of pathogen's survival in a host and the host's ability to induce protection against the pathogen. In this follow-up study, we document the impact of mutations on the pathogen's global protein expression and the influence of protein abundance on a mutant's attenuation and protection of vertebrate host against infection. iTRAQ labeling and mass spectrometry analysis of *E. chaffeensis* wildtype and mutants identified 564 proteins covering about 63% of the genome. Mutation in ECH_0379 gene encoding for an antiporter protein, causing attenuated growth in vertebrate hosts, led to overexpression of p28 outer membrane proteins, molecular chaperons, and metabolic enzymes, while a mutation downstream to the ECH_0490 gene that caused minimal impact on the pathogen's *in vivo* growth resulted in major changes in the expression of outer membrane proteins, transcriptional regulators and T4SS proteins. ECH_0660 gene mutation, causing the pathogen's rapid clearance and offering protection against wild type infection challenge in a vertebrate host, had a minimal impact on proteome similar to our prior observations from transcriptome analysis. While the global proteome data revealed fewer translated proteins compared to the transcripts identified from RNA deep sequencing analysis, there is a great deal of correlation noted between the global proteome and transcriptome analysis. Further, global proteome analysis, including the assessment of 2D resolved total and immunoproteomes revealed greater variations in the highly immunogenic p28-Omp proteins.

## Introduction

*Ehrlichia chaffeensis* is the etiological agent for human monocytic ehrlichiosis. It is a tick-borne pathogen causing infections also in dogs and other vertebrate hosts from *Amblyomma americanum* ticks (Dumler et al., 1993; Walker and Dumler, 1996; Davidson et al., 2001; Paddock and Childs, 2003). Mutations at certain genomic locations of *E. chaffeensis* affect the pathogen's ability of infection and persistence in a host independent of causing major changes in global gene expression (Cheng et al., 2013, 2015). Pathogenesis-associated *E. chaffeensis* proteins are possibly highly active in a host microenvironment and their differential expression in response to host cell defense is known to occur (Kuriakose et al., 2011). Progress has been made toward identifying proteins crucial for *E. chaffeensis* survival in a host cell environment (Singu et al., 2006; Seo et al., 2008; Lin et al., 2011). However, to date only a few abundantly expressed proteins are identified as associated with pathogenesis. For instance, high throughput proteomic studies identified macrophage-and tick cell-specific p28 outer membrane proteins, Type IV secretion system (T4SS) effector proteins, tandem repeat proteins (TRP), and ankyrin proteins (Anks) (Singu et al., 2006; Seo et al., 2008; Zhu et al., 2009; Wakeel et al., 2011; Luo and McBride, 2012). Differential protein expression of major antigenic proteins and virulence proteins of T4SS in a virulent strain and its attenuated form was reported in *Ehrlichia ruminantium* (Marcelino et al., 2012, 2015). Identifying proteins involved in pathogenesis and virulence, and documenting their differential expression may aid in the discovery of new protein targets valuable for therapeutic interventions and vaccine development against HME and in other related pathogenic rickettsial diseases.

Genetically mutated intracellular pathogens are important resources for studying microbial pathogenesis, and also aid in the efforts of vaccine development (McClure et al., 2017). Our previous study demonstrated that insertion mutations within the coding regions of ECH_0230, ECH_0379, and ECH_0660 genes resulted in the pathogen's attenuated growth, while mutation downstream to the ECH_0490 gene had no impact in its replication in vertebrate hosts (Cheng et al., 2013, 2015). Further, gene function disruption mutations within the genes ECH_0379 and ECH_0660 offered partial and complete protection against infection in a vertebrate host, respectively, while mutation in ECH_0230 offered no protection (Cheng et al., 2013, 2015; Nair et al., 2015; McGill et al., 2018). Our recent RNA deep sequencing study reported that mutation in ECH_0379 gene that codes for an anti-porter protein caused drastic down-regulation of several genes (Kondethimmanahalli and Ganta, 2018). While a mutation downstream to the ECH_0490 gene caused enhanced transcription from stress response genes, which possibly helped the mutant to better survive in vertebrate hosts. Mutation within the ECH_0660 gene, encoding for a phage like protein, showed minimal transcriptional variations compared to the wildtype strain, thus keeping the integrity of its transcriptome similar to wildtype *E. chaffeensis* (Kondethimmanahalli and Ganta, 2018). While we predict that the transcriptome data are suggestive of protein expression variations, additional experimental validation at the protein level is needed to confirm the results.

In this follow-up study, we used 2D proteome, 2D immunoproteome, and shotgun proteome deep sequencing methods to generate proteome data to assess if the proteomes are similarly altered in response to the mutations at certain genomic locations of *E. chaffeensis*. We present data in defining changes in the proteomes of the pathogen and discuss how the mutations may have impacted the protein expressions at global level.

## Materials and Methods

### Infection and Purification of *E. chaffeensis* From Infected Macrophages

*E. chaffeensis* Arkansas isolate wildtype and the mutants having mutations in ECH_0379, ECH_0490, and ECH_0660 genes were grown in the canine macrophage cell line, DH82 (Singu et al., 2006; Cheng and Ganta, 2008). Purification of host cell-free *E. chaffeensis* was carried out as we described earlier (Kondethimmanahalli and Ganta, 2018). Briefly, cultures having about 80–90% infection were harvested and resuspended in 1x phosphate buffered saline (PBS) and homogenized by passing through a 23 g needle for about 15–20 times. The lysate was centrifuged at 500x g for 5 min at 4°C and the supernatant was collected and filtered through a 2 μm sterile membrane filter (Millipore, Billerica, MA). The filtrate containing cell-free *Ehrlichia* was centrifuged at 15,000x g for 15 min. The pellet was resuspended in PBS, layered onto 30% Renografin solution (Mallinckrodt Inc, St. Louis, MO) and centrifuged at 100,000x g at 4°C for 1 h. The pellet of cell-free *Ehrlichia* were resuspended in PBS, centrifuged at 15,000x g for 15 min and the final pellet was used for protein extraction.

### Confocal Microscopy

A fraction of cell-free *Ehrlichia* organisms were transferred to a slide by cytocentrifugation, fixed with 4% paraformaldehyde for 15 min. Cell membrane was permealized by treating with 0.1% triton-X-100 and then blocked with 3% bovine serum albumin (BSA) for 30 min at room temperature to reduce non-specific staining. *Ehrlichia* organisms were incubated overnight at 4°C with *E. chaffeensis* specific anti-p28 OMP monoclonal antibody, 18.1 (Winslow et al., 2000) (1:400 dilution) or DnaK antibodies (Zhang et al., 2013) (1:500 dilution). Subsequently, the slides were incubated for 1 h at room temperature with Alexa Flour 594 goat-antimouse or anti-rabbit secondary antibodies (Molecular Probes, Eugene, OR), respectively. Uninfected DH82 cells and infected DH82 cells transferred to slides similarly and used by incubating with secondary antibodies alone to serve as negative controls. The cells in the negative control slides were stained with DAPI Fluoromount (Southern Biotech, Birmingham, AL) before examination by confocal microscopy. All slides were examined with a Carl Zeiss 700 laser scanning confocal microscopy (Carl Zeiss Optronics GmbH, Germany) and images were captured and analyzed using ZEN software.

### Protein Extraction

The proteome of wildtype and mutant organisms were extracted from independent replicates and the protein samples were separated into two portions, one of which was used for 2D gel experiment and the other for quantitative shotgun proteome analysis. The comprehensive protein expression data was compared between replicates of the same group of wildtype and mutants and then by comparing wildtype with the mutants. Supplementary Figure 1 has the workflow of sample preparation, LC-MS/MS analysis and proteins quantitation of *E. chaffeensis* wildtype and mutants organisms. Purified cell-free *Ehrlichia* were resuspened in lysis buffer containing 8M urea, 2M thiourea, 4% CHAPS, and protease inhibitors (Roche, Indianapolis, IN). Samples were sonicated on ice for 30 s using sonic dismembrator (Fisher Scientific, Hampton, NH) and centrifuged at 15,000x g for 15 min at 4°C. Proteins were precipitated and purified using a Readyprep 2D cleanup kit (BioRad, Hercules, CA) and then quantified using a detergent-compatible Bradford protein assay kit (BioRad, Hercules, CA). The proteins were reduced and alkylated using 10 mM DTT and 40 mM IAA, respectively and diluted 7 times with 100 mM TEAB.

### 2D Gel Electrophoresis and 2D Western Blot Analysis

One hundred μg of proteins were rehydrated on 11 cm, pH 4–7 linear IPG strips and then subjected to isoelectric focusing (IEF) using a Protean IEF Cell (Bio-Rad, Hercules, CA) using the conditions 250 V for 20 min, 8,000 V for 2 h and then 8,000 V till total volt hours reached 40,000. After IEF, the strips were reduced and alkylated sequentially for 15 min. The second-dimension SDS-PAGE was performed using 4–20% in a Criterion polyacrylamide gradient gels (Bio-Rad, Hercules, CA) for 2 h at 100 V. The 2-D gels were fixed in 50% methanol and 10% acetic acid and then stained with fluorescent SYPRO Ruby protein gel stain and scanned using Typhoon Trio Imager (GE Healthcare, Piscataway, NJ, USA). The 2D resolved total proteome gels were compared using the PD Quest^TM^ Advanced 2-D Analysis software, Version 8.0.1 (BioRad, Hercules, CA). The 2D gels were also used for Western blot analysis. The proteins resolved on 2D gels were transferred to a nitrocellulose membrane and the membrane was incubated with *Ehrlichia* specific polyclonal sera or with p28 OMP monoclonal antibody, 18.1 (Winslow et al., 2000). Polyclonal sera were obtained from wildtype C57BL6 mice infected with *E. chaffeensis* to detect immunological proteins (Ganta et al., 2004). The membranes were developed using X-ray developer.

### Peptide Fractionation and Tandem Mass Spectrometry Analysis

Proteins were digested with trypsin (Promega, Madison, WI) at an enzyme to protein ratio of 1:40 for 16 h at 37°C. Peptides were desalted with a Strata X C18 column (Phenomenex, Torrance, CA) and vacuum-dried according to the manufacturer's protocol. The peptides were labeled with iTRAQ reagents-8plex kit (Applied Biosystems, Foster City, CA) according to the manufacturer's protocol. The labeled peptides with different iTRAQ reagents were pooled, desalted, vacuum-dried and then used for peptide fractionation. The labeled peptides were reconstituted with buffer A containing 5% acetonitrile (ACN) in water and loaded onto a C18 column coated with 5 μm particles (Phenomenex, Torrance, CA). The peptides were separated on a Shimadzu LC-20AB HPLC pump system coupled with a high pH reverse phase column at a flow rate of 1 mL/min with a gradient of 5% buffer B (95% ACN in water) for 10 min, 5–35% for 40 min, and 35–95% for 1 min. Elution was monitored by measuring absorbance at 214 nm, and peptide fractions were collected in interval of 1 min. The eluted peptides were pooled into 10 fractions and vacuum-dried. Each fraction was resuspended in 2% ACN and 0.1% formic acid (FA) and centrifuged at 20,000 g for 10 min. The supernatant was then loaded onto a C18 trap column and separated at the flow rate of 5 μL/min for 8 min using a LC-20AD nano-HPLC instrument (Shimadzu, Kyoto, Japan). The peptides were eluted from trap column and separated by C18 column packed in-house. The gradient was run at 300 nL/min starting from 8 to 35% of buffer containing 2% H2O and 0.1% FA in ACN in 35 min, 60% for 5 min, 80% for 5 min, then 5% in 10 s and equilibrated for 10 min. The peptides separated from nano HPLC were subjected to Q EXACTIVE tandem mass spectrometry (Thermo Fisher Scientific, San Jose, CA). The data was acquired using a DDA (data dependent acquisition) method. The parameters set for MS analysis are: precursor scan range: 350–1,600 m/z at resolution of 70,000; MS/MS fragment scan range: >100 m/z at resolution of 17,500 in HCD mode; normalized collision energy setting: 27%.

### Protein Identification and Quantitation

The raw MS/MS data was converted into MGF format using Proteome Discoverer (Thermo Scientific, San Jose, CA) and then MGF files were searched against *E. chaffeensis* Arkansas isolate complete genome, accession: NC_007799.1 using Mascot 2.3 (Matrix Science, Boston, MA). The parameters are as follows: enzyme; trypsin, fragment mass tolerance; 0.05 Da, Mass values; monoisotopic, variable modifications; oxidation (M), iTRAQ8plex (Y), fixed modifications; carbamidomethyl (C), iTRAQ8plex (N-term), iTRAQ8plex (K), peptide mass tolerance; 20 ppm. IQuant automated software was used to quantify the labeled peptides (Wen et al., 2014). The protein quantitation pipeline included the following steps: protein identification, tag impurity correction, data normalization, missing value imputation, protein ratio calculation, and statistical analysis. To assess the confidence of peptides, the peptide-spectrum matches (PSMs) were pre-filtered at FDR of 1% and then, based on the parsimony principle, identified peptide sequences were assembled into a set of confident proteins (Savitski et al., 2015). In order to control the rate of false-positives at protein level FDR was set at <1% and the *p*-value was set less than 0.05. Hyper geometric test was used to obtain gene ontology (GO) terms.

### Quantitative Real Time-PCR

SYBR green detection based quantitative Real Time RT-PCR assays (SyBr qPCR) were performed to compare the gene expression to protein expression observed in the proteomics data. SuperScript III Platinum SYBR Green One-Step qRT-PCR Kit (Invitrogen, Carlsbad, CA) was used to measure the gene expression as described in Kondethimmanahalli and Ganta, 2018). Briefly, primer sets were designed for five randomly selected genes ECH_1142, ECH_1136, ECH_1130, ECH_0040, and ECH_0383 and used in the SyBR green qPCR assays to compare the wildtype, ECH_0379, ECH_0490, and ECH_0660 mutants (primer sets used for these genes are listed in Supplementary Table 1. RNA was normalized using 16S rRNA among different RNA batches and delta-delta Ct (ΔΔCt) calculation was employed to calculate relative change in the expression (Sirigireddy and Ganta, 2005).

## Results

### Two-Dimensional Proteins Mapping of *E. chaffeensis* Wildtype and Mutants Organisms

**Figure 1:**
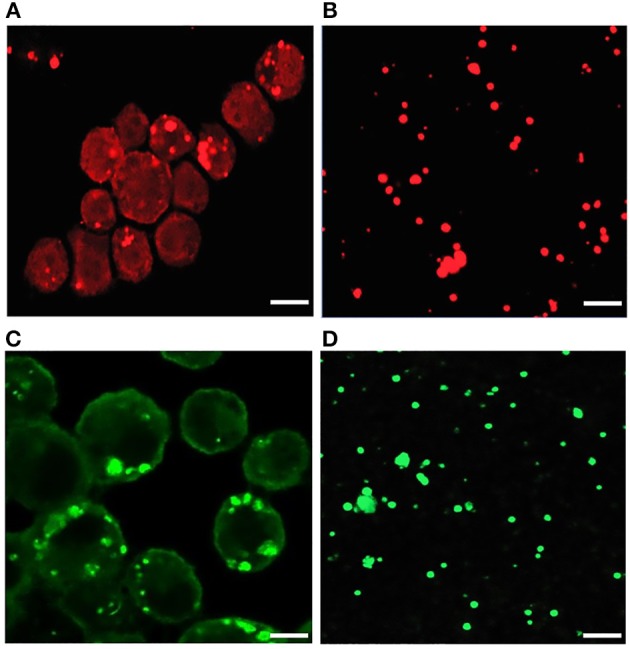
Confocal microscopy images of infected *E. chaffeensis* in DH82 cells and purified cell-free *Ehrlichia* organisms. About 80–90% infected DH82 culture was used in the experiment where *E. chaffeensis* specific anti-p28 OMP monoclonal antibody **(A)** and DnaK polyclonal sera **(B)** was used. Similarly, cell-free purified bacteria were immunolabeled with p28-OMP monoclonal **(C)** and DnaK polyclonal sera **(D)**. (Scale bar, 10 μm).

**Figure 2:**
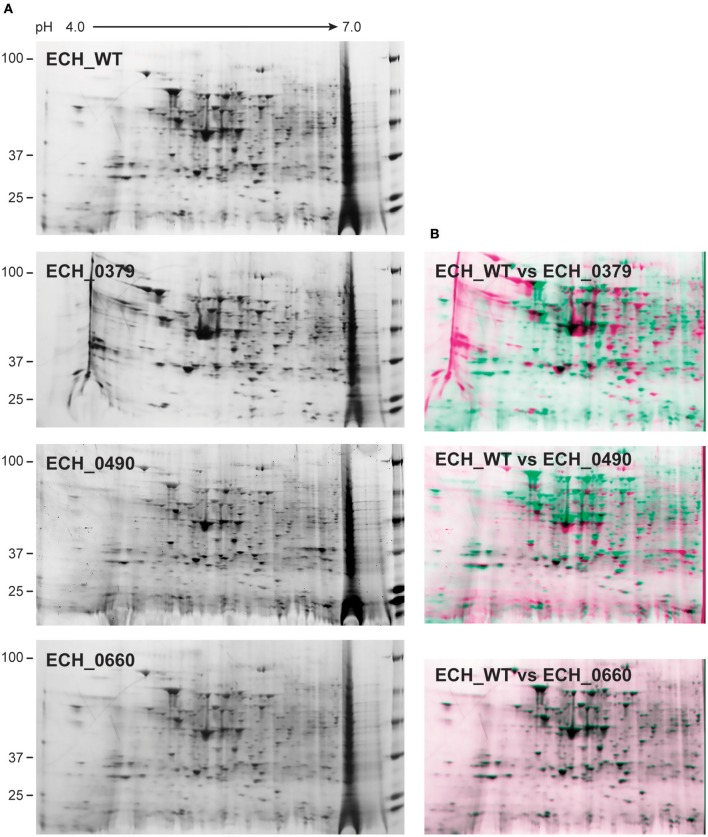
2D gel resolved protein expression maps of *E. chaffeensis* wildtype and its three mutants. **(A)** One hundred μg of proteins were separated on 11 cm, pH 4–7 linear IPG strips and second dimension SDS-PAGE was performed using 4–20% Criterion gels. Gels were stained with SYPRO Ruby protein stain. The pH and molecular weights of the proteins are shown on the top and left side of the gel images, respectively. **(B)** The 2D proteomes of mutants were compared with the wildtype *E. chaffeensis* proteome using the PD Quest^TM^ Advanced 2-D Analysis software where we observed 89% match between wildtype and ECH_0660, followed by ECH_0490 having matched at 64% and the lowest matching of 55% for the ECH_0379 with the wildtype *E. chaffeensis* proteome.

**Figure 3:**
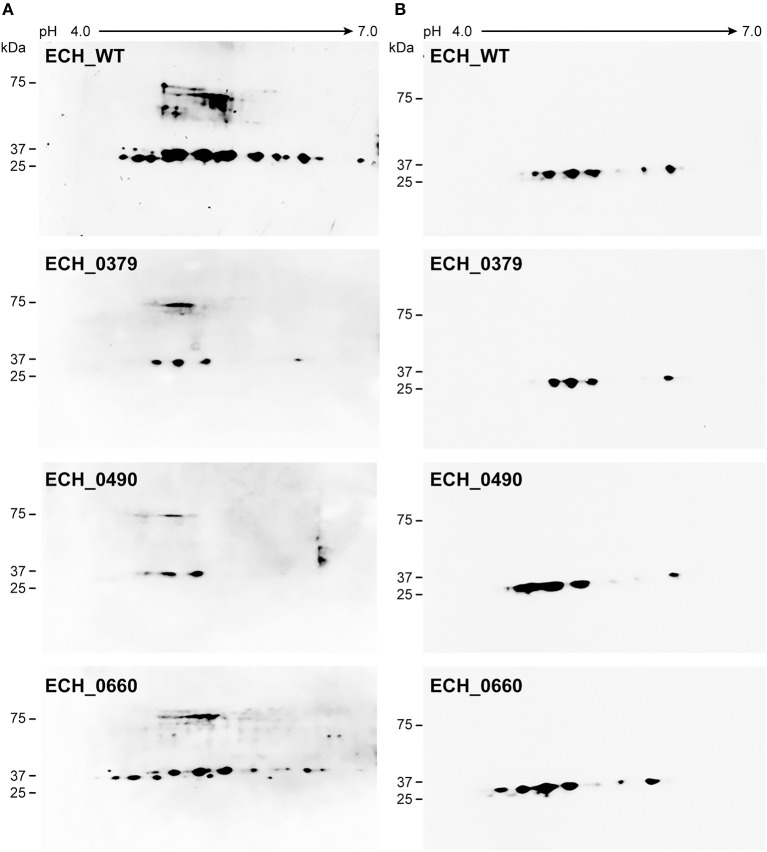
*E. chaffeensis* wildtype and mutants' proteins immunoblotted with polyclonal sera or with p28 monoclonal. One hundred μg of proteins each were separated on 11 cm, pH 4–7 linear IPG strips, and SDS-PAGE was performed using 4–20% Criterion gels. The resolved proteins from gels were transferred to nitrocellulose membranes and subjected to Western blot analysis with *E. chaffeensis* polyclonal sera **(A)** or with p28 OMP monoclonal antibody **(B)**.

Our recent transcriptome assessment of wildtype and three mutant strains of *E. chaffeensis* aided in gaining considerable understanding of how mutations at certain genomic sites impact global gene expression changes, while other having minimal impact. Further, the transcriptome study provided novel insights regarding how a specific mutation leading to attenuation may help a host in developing a protective host response (Kondethimmanahalli and Ganta, 2018). To assess if proteome data of wildtype and mutant strains of *E. chaffeensis* correlate well to the observed transcriptome changes, we first compared their protein profiles by 2D gel analysis. *E. chaffeensis* organisms were first purified free of host cells by employing multiple purification steps (Kondethimmanahalli and Ganta, 2018). The purity of the cell-free organisms was monitored by confocal fluorescence microscopy (Figure 1). Subsequently, total bacterial proteins were recovered and used to generate 2D proteome maps using narrow range pH strips (pH 4–7) to facilitate a better proteome separation (Celis and Gromov, 1999). Majority of the proteins resolved on 2D gels have molecular weights and pH ranged from 15 to 100 kDa and pH 5–7, respectively (Figure 2). Consistent with our prior transcriptome analysis (Kondethimmanahalli and Ganta, 2018), we observed greater homology for the 2D resolved proteomes of wildtype and ECH_0660 mutant (89% matched) and relatively less correlation observed between the wildtype and ECH_0490 (64% matched), while negative correlation observed for the ECH_0379 with the wildtype proteomes (55% matched) (Figure 2). We subsequently performed immunoblot analysis of the 2D resolved proteins using a murine host derived *E. chaffeensis-*specific polyclonal sera (Figure 3A and Supplementary Figures 2A,B) or with a p28 Omp gene-specific monoclonal antibody (p28-Omp MAb) (Figure 3B and Supplementary Figures 2C,D). Independent of the polyclonal sera or p28-Omp MAb used, the major immunoreactive proteins observed were in the region predicted to be in the region spanning to the p28-Omp region independent of the polyclonal sera or the p28-Omp MAb were used (Figures 3A,B). These observations suggest that the major immunogenic antigens of the bacterium are the p28-Omps. Further, the MAb detected multiple resolved proteins on a pH gradient within the molecular weight ranged from 28 to 30 kDa. This is independent of the protein fractions used from mutants or from wildtype organisms (Figure 3A). While we did not see much variation in replicates of each strain, wildtype and ECH_0660 mutant had very similar 2D immunoblots, while, mutant-specific variations in the immunogenic p28-Omp proteins are more notable for ECH_0379 and ECH_0490 compared to the wildtype (Figure 3).

### Comprehensive Analysis of *E. chaffeensis* Global Proteome

To improve the proteome wide coverage and also to identify large numbers of expressed proteins, we performed global shotgun proteome analysis of *E. chaffeensis* wildtype and mutant organisms. We carried out iTRAQ labeling and Q EXACTIVE mass spectrometry analysis, which led to the confirmed identification of 564 proteins representing 63% of the total predicted ORFs from the *E. chaffeensis* genome. We observed a two-fold increase in the total number of proteins identified in this study compared to our previous proteome analysis, where only 278 proteins were detected in *E. chaffeensis* cultured in macrophages (Seo et al., 2008) (Supplementary Table 2). Comprehensive proteome analysis of the wildtype *E. chaffeensis* is also reported by Lin et al. (2011). The authors reported 1,021 *E. chaffeensis* proteins, representing a total of 92% of the predicted proteins of the pathogen. Our identified proteins are 39% fewer compared to the total numbers of transcripts (920) detected by next generation RNA Seq analysis (Kondethimmanahalli and Ganta, 2018). Similar to the 2D resolved proteomes, the greatest correlation was observed for wildtype with ECH_0660 mutant (94% matched), followed by ECH_0490 (88%) and the least correlation was observed for ECH_0379 (74%).

**Figure 4:**
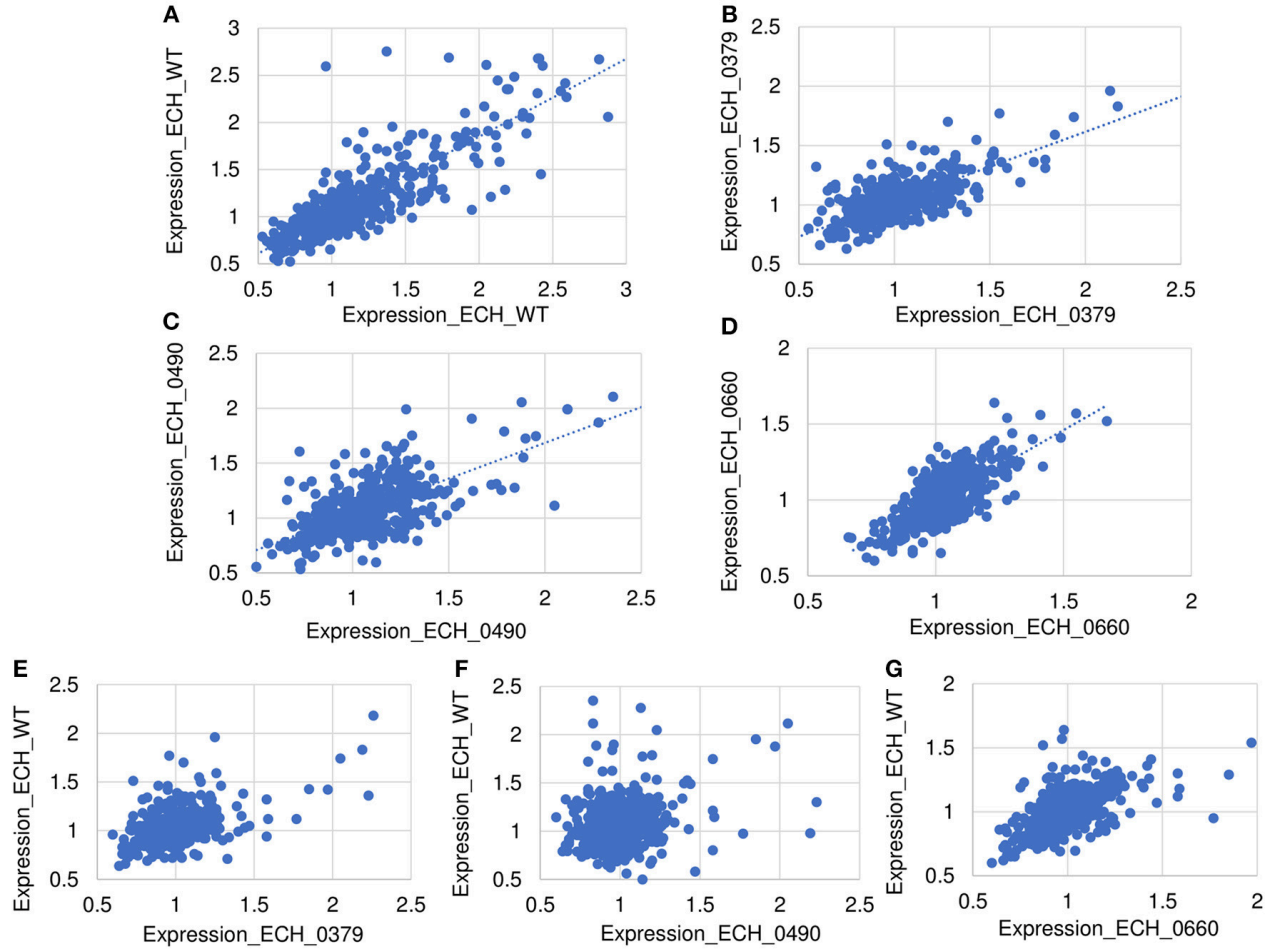
Scatter plot of protein expression data. Scatter plots of protein expression data for replicates was presented for *E. chaffeensis* wild-type **(A)** and mutants ECH_0379 **(B)**, ECH_0490 **(C)**, and ECH_0660 **(D)**, which revealed a higher degree of correlation. Scatter plots of protein expression data for *E. chaffeensis* wildtype vs. mutant ECH_0379 **(E)**, wildtype vs. mutant ECH_0490 **(F)**, which revealed a lower degree of correlation, whereas the wildtype vs. ECH_0660 **(G)** had a higher level of correlation.

About 81% of the total identified proteins spanned the molecular weights ranging from 10 to 60 kDa, whereas 2% of the proteins represented proteins having molecular weights <10 kDa and 16% belonging to the molecular weights >60 kDa (Supplementary Figure 3A). We compared the predicted molecular weights of proteins derived from genome sequence with observed experimentally elucidated molecular weights from proteomics data (Supplementary Figure 3B). There were no significant differences in molecular weight distribution while majority of proteins have molecular weights in the range of 10–60 kDa in both datasets. About 73% of proteins identified included more than one peptide; 14.3, 8.5, and 13.8% of the proteins mapped comprised of 2, 5 and >10 peptides, respectively (Supplementary Figure 3C). About 63% of the detected proteins had >10% sequence coverage (Supplementary Figure 3D). Supplementary Table 3 lists proteins and their sequence coverage (%), number of unique peptides, and number of unique spectrum used for protein identification in the wildtype and in all three mutant organisms. The protein abundances between biological replicates were compared to demonstrate the reproducibility of the wildtype and mutants' proteomes (Figure 4). The scatter plot of protein expression data from replicates samples of wildtype (Figure 4A) and mutants ECH_0379 (Figure 4B), ECH_0490 (Figure 4C), and ECH_0660 (Figure 4D) showed a high degree of protein expression correlation. The protein expression of wildtype vs. ECH_0379 (Figure 4E) and wildtype vs. ECH_0490 (Figure 4F) showed a negative correlation. Notably, the expression plot of wildtype vs. ECH_0660 had a greater correlation that is similar to replicates within wildtype or the respective mutant strains (Figure 4G). Hypothetical proteins of unknown function were the highest expressed in the list of detected proteins. The proteins were functionally categorized with references to the annotated genome data (Hotopp et al., 2006) (Supplementary Table 2). Majority of the expressed proteins were represented in the groups belong to protein synthesis, protein turnover, cellular processes, energy metabolism, and transport followed by regulatory functions, amino acid and nucleotide biosynthesis and cell envelop proteins (Supplementary Table 2).

### ECH_0379 Mutation Caused Differential Expression of Outer Membrane Proteins, Chaperons, and Metabolic Enzymes

**Figure 5:**
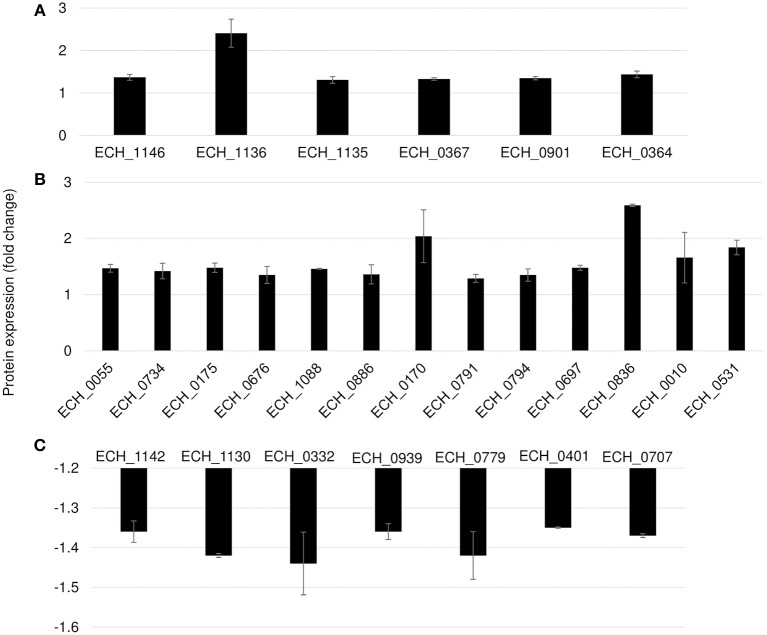
Differentially expressed proteins in mutant ECH_0379. **(A)** p28 OMP's and chaperons as upregulated, **(B)** metabolic enzymes and hypothetical proteins as upregulated, **(C)** p28 OMP's and metabolic enzymes as downregulated. Protein expression was determined by comparing the expression values of mutant ECH_0379 and wildtype. The GenBank numbers are listed in Table 1. Fold changes were considered significant with < 1% FDR at protein level and with a *p*-value was than 0.05.

Differential protein expression was determined by comparing the expression values of mutants and wildtype. Fold changes were considered significant with a *p*-value < 0.05 and false discovery rate (FDR) <1%. Twenty-five housekeeping gene products representing ribosomal protein complex proteins were randomly selected (Supplementary Table 4) to assess if their expression is similar for the wildtype and the three mutants; indeed there was no significant difference in their expression levels. Based on these criteria, 19 genes were identified as predominantly upregulated and 7 genes were downregulated in the ECH_0379 mutant compared to wildtype (Figure 5; Table 1). These differentially expressed proteins included outer membrane proteins, chaperons, and metabolic enzymes. Three p28-Omps; OMP-p28-2 (ECH_1146), OMP-1B (ECH_1136), and OMP-1A (ECH_1135) and three chaperon proteins; ATP-dependent chaperone ClpB (ClpB) (ECH_0367), ATP-dependent Clp protease proteolytic subunit (ClpP) (ECH_0901) and heat shock protein 10 (GroES) (ECH_0364) were also among the upregulated in mutant ECH_0379 compared to wildtype (Figure 5A). Several metabolic enzymes; phosphoglycerate kinase (ECH_0055), peroxidase (ECH_0734), malate dehydrogenase (ECH_0175), bifunctional ornithine acetyltransferase/N-acetylglutamate synthase (ECH_0676), ATP synthase subunit (ECH_1088), acetylornithine transaminase (ECH_0886), 5'-nucleotidase SurE (ECH_0791), and leucine-tRNA ligase (ECH_0794) were also upregulated in the ECH_0379 mutant (Figure 5B). Four hypothetical proteins (ECH_0697; ECH_0836; ECH_0010; ECH_0531) were also among the highly expressed and upregulated proteins. On the contrary, two p-28-Omps; OMP-1F (ECH_1142) and OMP-1X (ECH_1130) and four metabolic enzymes; phosphoglycolate phosphatase (ECH_0332), formylmethionine deformylase (ECH_0939) and phosphatidylserine decarboxylase proenzyme (ECH_0779) and protease (ECH_0401) were among the downregulated proteins (Figure 5C).

**Table 1:** Proteins differentially expressed in mutant ECH_0379.

**GenBank # CP000236.1**	**NCBI sequence # NC 007799.1**	**Protein**	**Fold change**
**UPREGULATED PROTEINS**			
ECH_1146	ECH_RS04635	OMP-p28-2	1.37
ECH_1136	ECH_RS04600	OMP_1B	2.41
ECH_1135	ECH_RS04595	OMP_1A	1.31
ECH_0367	ECH_RS01495	ATP-dependent chaperone ClpB (ClpB)	1.33
ECH_0901	ECH_RS03705	ATP-dependent Clp protease proteolytic subunit (ClpP)	1.35
ECH_0364	ECH_RS01480	Molecular chaperone GroES	1.44
ECH_0055	ECH_RS00260	Phosphoglycerate kinase	1.47
ECH_0734	ECH_RS03050	Peroxidase	1.42
ECH_0175	ECH_RS00740	Malate dehydrogenase	1.48
ECH_0676	ECH_RS02840	Ornithine acetyltransferase/N-acetylglutamate synthase	1.35
ECH_1088	ECH_RS04415	ATP synthase subunit B	1.46
ECH_0886	ECH_RS03645	Acetylornithine transaminase	1.36
ECH_0170	ECH_RS00715	variable length PCR target protein	2.04
ECH_0791	ECH_RS03285	5′-nucleotidase SurE	1.29
ECH_0794	ECH_RS03300	Leucine–tRNA ligase	1.35
ECH_0697	ECH_RS02910	Hypothetical protein	1.48
ECH_0836	ECH_RS03455	Hypothetical protein	2.59
ECH_0010	ECH_RS00065	Hypothetical protein	1.66
ECH_0531	ECH_RS02210	Hypothetical protein	1.84
**DOWNREGULATED PROTEINS**			
ECH_1142	ECH_RS04620	OMP-1F	−1.36
ECH_1130	ECH_RS04570	OMP-1X	−1.42
ECH_0332	ECH_RS01355	Phosphoglycolate phosphatase	−1.44
ECH_0939	ECH_RS03835	Formylmethionine deformylase	−1.36
ECH_0779	ECH_RS03230	Phosphatidylserine decarboxylase proenzyme	−1.42
ECH_0401	ECH_RS01630	Protease	−1.35
ECH_0707	ECH_RS02960	Hypothetical protein	−1.37

### ECH_0490 Mutation Caused Differential Expression of Outer Membrane Proteins and Transcriptional Regulator

**Figure 6:**
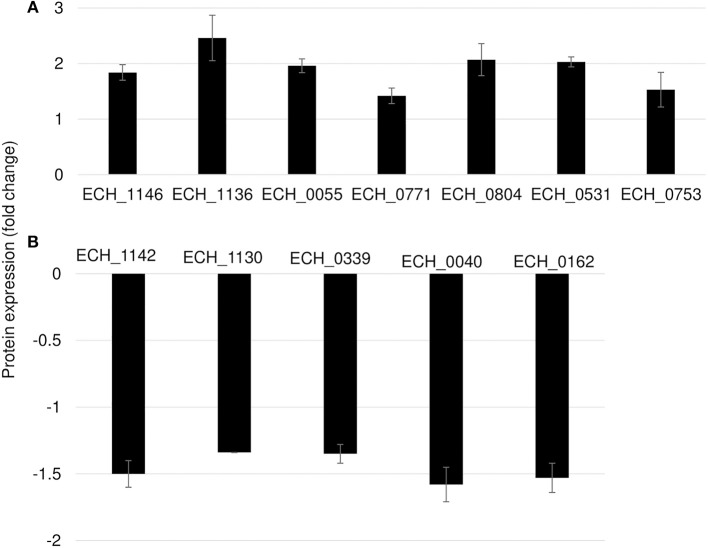
Differentially expressed proteins in mutant ECH_0490. **(A)** Upregulated proteins, **(B)** downregulated proteins. Protein expression was determined by comparing the expression values of mutant ECH_0490 and wildtype. The GenBank numbers are listed in Table 2. Fold changes were considered significant with < 1% FDR at protein level and with a *p*-value was than 0.05.

In this mutant, two proteins belong to the p28-Omp family; OMP-p28-2 (ECH_1146), OMP_1B (ECH_1136) and phosphoglycerate kinase (ECH_0055), NAD-glutamate dehydrogenase (ECH_0771), and DNA-binding protein (ECH_0804) were among the upregulated proteins (Figure 6A; Table 2). Whereas, OMP-1F (ECH_1142), OMP-1X (ECH_1130), sigma-54-dependent Fis family transcriptional regulator (ECH_0339), T4SS protein VirD4 (ECH_0040), and DNA-binding integration host factor, alpha subunit (ECH_0162) were in the downregulated proteins' list (Figure 6B).

**Table 2:** Proteins differentially expressed in mutant ECH_0490.

**GenBank #CP000236.1**	**NCBI sequence #NC_007799.1**	**Protein**	**Fold change**
**UPREGULATED PROTEINS**			
ECH_1146	ECH_RS04635	OMP-p28-2	1.84
ECH_1136	ECH_RS04600	OMP_1B	2.46
ECH_0055	ECH_RS00260	Phosphoglycerate kinase	1.96
ECH_0771	ECH_RS03195	NAD-glutamate dehydrogenase	1.42
ECH_0804	ECH_RS03340	DNA-binding protein	2.07
ECH_0531	ECH_RS02210	Hypothetical protein	2.03
ECH_0753	ECH_RS03115	Hypothetical protein	1.53
**DOWNREGULATED PROTEINS**			
ECH_1142	ECH_RS04620	OMP-1F	−1.5
ECH_1130	ECH_RS04570	OMP-1X	−1.34
ECH_0339	ECH_RS01385	Sigma-54-dependent transcriptional regulator	−1.37
ECH_0040	ECH_RS00205	Type IV secretion system protein VirD4	−1.58
ECH_0162	ECH_RS00680	DNA-binding integration host factor, alpha subunit	−1.53

### Mutation in ECH_0660 Gene Led to Minimal Proteome Alterations

**Figure 7:**
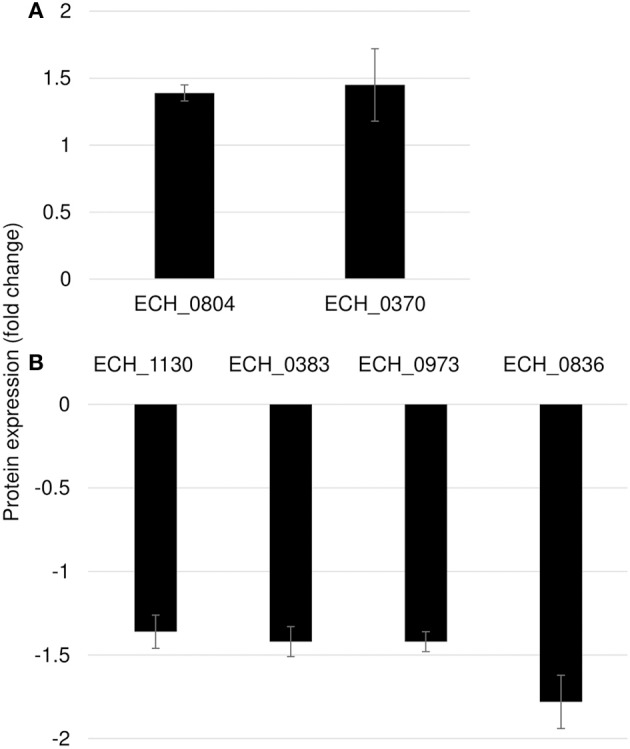
Differentially expressed proteins in mutant ECH_0660. **(A)**, upregulated proteins; **(B)**, downregulated proteins. Protein expression was determined by comparing the expression values of mutant ECH_0660 and wildtype. The GenBank numbers are listed in Table 3. Fold changes were considered significant with < 1% FDR at protein level and with a *p*-value was than 0.05.

We observed only six genes as notably differentially expressed in this mutant (Figure 7; Table 3). They included DNA-binding protein HU (ECH_0804) and the leucyl aminopeptidase protein (ECH_0370) as upregulated genes, whereas the OMP-1X (ECH_1130), type I secretion system (T1SS) permease (ECH_0383) and cytochrome b (ECH_0973) were among the downregulated. We observed OMP-p28-2, OMP_1B were commonly upregulated, whereas OMP-1F and OMP-1X were commonly down-regulated proteins in ECH_0379 and ECH_0490 compared to wildtype (Supplementary Table 5). Omp proteins' expressions showed no significant difference in ECH_0660 mutant expect the down-regulation of OMP-1X.

**Table 3:** Proteins differentially expressed in mutant ECH_0660.

**GenBank #CP000236.1**	**NCBI sequence #NC_007799.1**	**Protein_ID**	**Fold change**
**UPREGULATED PROTEINS**			
ECH_0804	ECH_RS03340	DNA-binding protein	1.39
ECH_0370	ECH_RS01510	Leucyl aminopeptidase	1.45
**DOWNREGULATED PROTEINS**			
ECH_1130	ECH_RS04570	OMP-1X	−1.36
ECH_0383	ECH_RS01555	Type I secretion system permease	−1.42
ECH_0836	ECH_RS03455	Hypothetical protein	−1.42
ECH_0973	ECH_RS03980	Cytochrome b	−1.78

### Correlation Between Protein Expression and RNA Expression

**Figure 8:**
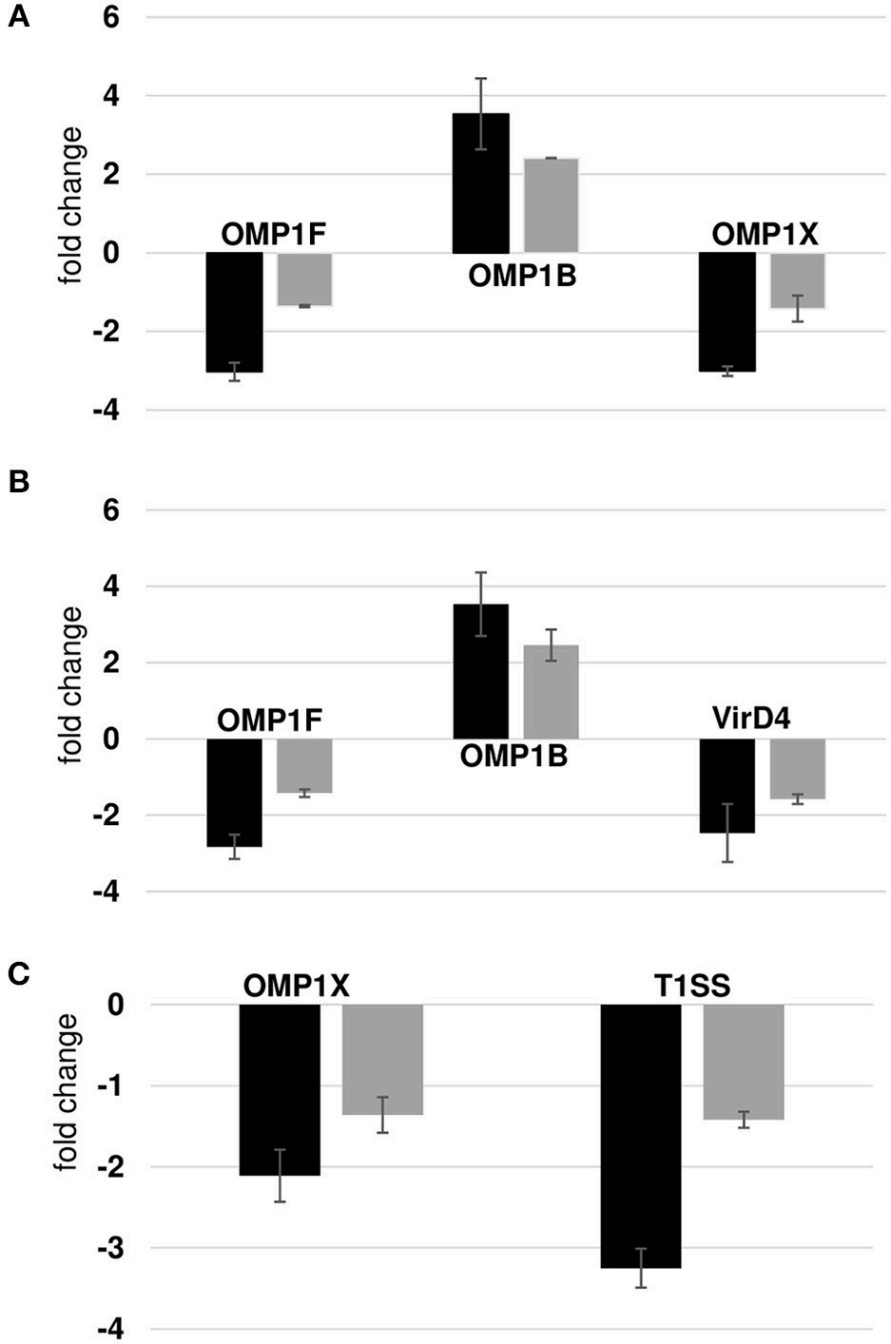
Correlation between protein expression and RNA expression. Fold changes in wildtype vs. ECH_0379 mutant **(A)** or ECH_0490 mutant **(B)** or ECH_0660 mutant **(C)** were assessed by RT-PCR. Black bars represent qPCR data and gray green bars represents proteomics data.

We compared our prior published RNA deep sequencing data to the current proteome data. A combined total of 920 transcripts were detected from wild-type and the three mutants from RNA deep sequencing analysis, while only 564 proteins were confirmed from the proteome data. We observed a significant overlap among the prominent group of expressed genes in both data sets. For instance, we detected all p28 family proteins and majority of ribosomal proteins, chaperone proteins in both the dataset suggesting the overall correlation between the two data sets was modest. On the contrary, the discordance between transcript and protein abundance was high in hypothetical proteins and T4SS proteins. We detected 71 and 188 hypothetical proteins in proteomics and RNA-Seq data, respectively. Three and 13 T4SS proteins were detected in proteomics and RNA-Seq data, respectively. The gaps in our proteomic data set were apparent since identification of low abundant proteins by shotgun proteomics is still a major challenge, although the possibility that some of the transcribed genes are not translated as proteins cannot be ruled out. Complexity of biological regulation of proteome and transcriptome and posttranslational modification of proteins also contribute to low correlation between two data sets. In general, transcripts level does not usually equal the protein level; the data obtained by either approach must be evaluated independently. Nevertheless, p28 family proteins identified in the proteome showed similar abundance pattern in the transcriptome. To verify these correlations, we also performed RT-PCR validation assays on a few randomly selected gene transcripts (Figure 8). Transcripts for three proteins belonged to p28-Omp gene family (OMP-1B, OMP-1F, and OMP-1X), a virulence associated gene, VirD4, and T1SS permease was evaluated. OMP-1B and OMP-1F were up and down regulated in mutants ECH_0379 and ECH_0490, respectively according to the proteomics data and the qPCR showed similar trend of gene expression (Figures 8A,B). OMP-1X was down regulated in ECH_0379 and ECH_0660 gene mutants and qPCR data similarly showed down regulation of the transcripts of these two genes (Figures 8A,C). In ECH_0490 mutant strain, VirD4 protein down regulation was well correlated with its mRNA expression, similarly in ECH_0660 mutant T1SS protein down regulation was also well correlated with its mRNA expression (Figures 8B,C).

## Discussion

In the present study, a total of 564 proteins were confirmed as expressed proteins in *E. chaffeensis*. Among these, about 80% had molecular weights ranged between 10 and 60 kDa; 73% of which were identified by more than two peptides and 63% proteins had sequence coverage >10%. Proteins identified from the shotgun proteomic analysis are about 50% more compared to the previous assessment by the 1D gel resolved and mass spectrometry analysis (Seo et al., 2008). Nonetheless, the detected proteins are 40% less than the transcripts detected by RNA deep sequencing assessment (Kondethimmanahalli and Ganta, 2018). These results suggest that the shotgun proteome methods remain less sensitive compared to the next generation of RNA Seq technology; regardless the proteome data are important in validating the predicted genes as functionally active (Hotopp et al., 2006). The proteome data included 91 hypothetical proteins with unknown function. As these were within the core proteome, we presume that they represent an important set of proteins for *E. chaffeensis* infection and replication.

The abundant expression of p28-Omps, molecular chaperones proteins, cytochrome c, and NADH oxidoreductase are among the commonly expressed in *E. chaffeensis*, independent of the wildtype or mutants, suggesting that they are among the list of essential proteins required for the bacterium. Similarly, several proteins involved in protein synthesis, energy metabolism, DNA metabolism, and transport mechanisms were identified which are also likely represent as the pool of housekeeping proteins. In our previous transcriptome study, we also observed similar trend of abundance in gene expression patterns (Kondethimmanahalli and Ganta, 2018) suggesting that these set of genes is ubiquitously expressed. The p28-Omp multigene locus includes 22 genes encoding for the bacterial membrane proteins (Ohashi et al., 1998; Reddy et al., 1998; Yu et al., 2000). These immunogenic proteins are likely essential for immune response and adhesion functions, as they are also widely reported as the predominantly expressed immunodominant proteins in several closely related *Ehrlichia* species (Kumagai et al., 2006; Singu et al., 2006; Seo et al., 2008; Marcelino et al., 2015). Indeed, our 2D immuneproteome analysis using the *E. chaffeensis* polyclonal sera or with a MAb confirms this hypothesis. Omps are important virulence factors in several Gram-negative bacterial pathogens and are used for host cell invasion (Ojogun et al., 2012; Martinez et al., 2014). We detected the abundance of p28-Omps on 2D gel and in addition we found all 22 p28-Omp family proteins in the shotgun proteomics, similar to RNA-Seq data (Kondethimmanahalli and Ganta, 2018; Supplementary Table 5). Our previous study documented macrophage and tick cell-specific expression of p28-Omps where Omp-p28 (p28-Omp 19) transcript was detected as abundantly expressed in macrophage culture derived *E. chaffeensis*, whereas the transcript for Omp-1B (gene 14) was the major expressed transcript for the bacterium cultured in tick cells (Ganta et al., 2009). Similarly, differential expression of membrane protein-encoding genes between the virulent and attenuated strains were observed using OMICS methods in *E. ruminantium* (Marcelino et al., 2015; Pruneau et al., 2018). Consistent with the prior studies, p28-Omps are among the most abundantly expressed and immunogenic proteins in both wildtype and in the three mutants assessed. Further, their expression patterns are also variable in the ECH_0379 and ECH_0490 mutants compared to the wildtype, however, the ECH_0660 mutant had a very similar expression patterns as observed for the wildtype. The expression abundance and variations in the expression from the p28-Omps among different mutants, coupled with their highly immunogenic nature, suggest that they are likely among the most important proteins in contributing to the pathogen's outer membrane architecture, thereby possibly influencing the pathogen's ability of adhesion and infection.

NADH dehydrogenase inhibits host cell apoptosis by countering the phagosomal NOX2 response (Lin and Rikihisa, 2007). Stress response proteins are important for *E. chaffeensis* (Kuczynska-Wisnik et al., 2017) and their abundant expression is possibly essential for cell homeostasis and oxidative stress response, as reported for other rickettsiales (Hajem et al., 2009; Lin et al., 2011). Stress response genes in transcriptome were similarly altered in *E. chaffeensis* suggesting that their gene products are critical for the *E. chaffeensis* stress response (Kondethimmanahalli and Ganta, 2018). Other abundantly expressed proteins included ATP synthase, RNA polymerases, elongation factor and preprotein translocase, which are among the house-keeping proteins involved in pathogen's protein synthesis, transcription, and cellular trafficking.

Mutation in ECH_0660 gene resulted in the pathogen's attenuated growth (Cheng et al., 2013, 2015), but displayed minimal transcriptome changes (Kondethimmanahalli and Ganta, 2018) and that is confirmed with the proteome change, whereas ECH_0379 mutation also resulted in the attenuated growth while resulting in major changes to transcriptome and similarly in proteome changes. Likewise, ECH_0490 gene had no impact in the pathogen's persistence in vertebrate hosts, but caused relatively more changes in the protein/transcriptome expression patterns as compared to the ECH_0660 mutant. As we stated in our previous transcriptome analysis paper (Kondethimmanahalli and Ganta, 2018), the rapid clearance of ECH_0660 mutant in vertebrate host may have resulted due to the essential nature of the gene product encoding for a phage like structure protein and from other genes located near to this gene, despite maintaining most of the transcriptome and proteome being similar to the wildtype *E. chaffeensis*. We are currently extending investigations in defining the importance of this gene product to *E. chaffeensis*. On the contrary, we reasoned that mutation in Ech_0379 (an antiporter protein gene) induced significant alterations to overcome the deficiency of this gene expression. As antiporter proteins are critical also for the bacterial pH homeostasis, the mutation also caused attenuated growth. The ECH_0490 mutation also causing relatively more changes to transcriptome, which also reflected at the proteome data in the current study. As this mutation is in the intergenic region, it is likely that there is no loss of gene expression from this gene and thus did not impact the pathogen's continued persistence in vertebrate hosts (Cheng et al., 2013, 2015). Because of the extensive conservation of gene expression in ECH_0660 similar to wildtype and that the likely essential nature of the gene product for the bacterial continued survival, this mutant could be recognized well by the host immune response as it is very similar to the wildtype at the proteome level, thus enabling the vertebrate hosts to recognize this mutant as closer to wildtype organism in inducing a stronger host response mimicking wildtype infection and also conferring protection against wild type infection challenge as we reported previously (Nair et al., 2015; McGill et al., 2016).

Mutation in ECH_0379 gene caused up regulation of 19 proteins and 7 proteins as down regulated. In contrast, transcriptome data from the next gen RNA Seq of this mutant revealed significantly more genes as down regulated (Kondethimmanahalli and Ganta, 2018). The differences in the proteome vs. transcriptome analysis may be a reflection of differences in the transcription vs. translation or alternatively, shotgun proteome methods may be less sensitive than the next gen RNA Seq methods. Nonetheless, proteome analysis is important in both validating the transcriptome analysis and also in confirming that the genes for which peptide fragments were detected are indeed functionally active genes. The abundant synthesis of p28-Omps and chaperons may also be important for the pathogen's survival strategy to defend the host immune response. The same trends of expression of Omps (Omp-1B, Omp-1F, and Omp-1X) were confirmed by RT-PCR as in proteome analysis for all three mutants. Several metabolic enzymes: phosphoglycerate kinase, peroxidase, malic enzyme, ornithine acetyltransferase/N-acetylglutamate synthase, ATP synthase subunit, acetylornithine transaminase, 5'-nucleotidase SurE, and leucine–tRNA ligase were also found to be overexpressed. In general, pathogenic bacteria use metabolic enzymes that undermine the host immune system so that they can defend the host (Eisenreich et al., 2013; Olive and Sassetti, 2016). Indeed, although ECH_0379 gene mutation resulted in the attenuated growth of the organism in both an incidental host (dog) and in the reservoir host (white-tailed deer; Cheng et al., 2013, 2015), it failed to offer complete protection against wildtype infection challenge (Nair et al., 2015). The down regulation of OMP-1F and OMP-1X and phosphoglycolate phosphatase, formylmethionine deformylase, and phosphatidylserine decarboxylase in ECH_0379 mutant suggesting the complex regulatory feedback mechanisms of same set of proteins; in this case p28 Omps and metabolic enzymes, thus making the host likely less effective in initiating a protective host response when exposed to the mutant organism (Nair et al., 2015).

ECH_0490 mutant had no impact in vertebrate hosts but caused more changes in the global protein expression patterns. The proteomic changes observed in this mutant may not have had any negative impact on the pathogen growth because this mutant grows similar to the wildtype both in white-tailed deer and in dogs (Cheng et al., 2013, 2015). The ECH_0490 mutant had increased abundance of two Omps, OMP-p28-2 and OMP_1B (ECH_1136) and two hypothetical proteins, ECH_0531 and ECH_0753. We also reported significant increase in mRNA levels of these genes suggesting a greater correlation between transcriptome and proteome data sets. Nevertheless, changes in the abundance of the outer membrane proteins may be associated with overall changes in the membrane architecture, thereby altering the pathogen's susceptibility to host defense (Lin et al., 2011).

We identified metabolic enzymes phosphoglycerate kinase, glutamate dehydrogenase as overexpressed, whereas proteins of secretary pathway such as VirD4, and transcriptional regulators, DNA-binding integration host factor alpha subunit and Sigma*-*54-dependent *Fis* family transcriptional regulator (sigma-54 Fis protein) were downregulated in mutant ECH_0490. We also observed overexpression of metabolic enzymes in the ECH_0379 mutant, which may affect host physiology and metabolic responses to infection. Effector proteins are known to alter the host cell gene expression and contribute to bacterial virulence (Gillespie et al., 2010; Sinclair et al., 2014; Lin et al., 2016; Yan et al., 2018). The downregulation of DNA-binding proteins and secretary pathway proteins observed in this study was well correlated with their mRNA expression documented by next generation RNA-Seq analysis (Kondethimmanahalli and Ganta, 2018). The ECH_0490 mutant grows similar to the wildtype pathogen both in white-tailed deer (the reservoir host) and in dogs (an incidental host) and also in its tick host, *Amblyomma americanum* (Cheng et al., 2013, 2015). Changes in the proteome of this mutant suggest that the mutation impacted protein expression and yet did not adversely affect the pathogen's survival in vertebrate and tick hosts (Cheng et al., 2013, 2015).

The minimal variation in the proteome of the ECH_0660 mutant compared to the wildtype *E. chaffeensis* was consistent with our prior study where we reported similar minimal impact at the transcriptome level (Kondethimmanahalli and Ganta, 2018). Notably, this mutant has a severe growth defect in vertebrate hosts and that the host response against it is strongest and is also sufficient in offering complete protection against wildtype pathogen infection challenges by needle infection and tick transmission infection challenges (Nair et al., 2015; McGill et al., 2016). Few proteins altered in this mutant included DNA-binding protein HU (ECH_0804) and the leucyl aminopeptidase protein as upregulated, whereas only one p28-Omp protein, OMP-1X (ECH_1130) was down regulated. Similarly, TISS permease and cytochrome b were down regulated. Quantitative real-time RT-PCR data showed similar downregulation of OMP-1X and TISS permease. The protein abundance variations were significantly less compared to other two mutants.

## Conclusions

The proteins identified by global proteome analysis covered about 63% of the genome. Mutation in ECH_0379 led to major changes in the proteome with overexpression of several p28 Omps, molecular chaperons, and metabolic enzymes, while mutation downstream to the ECH_0490 gene impacted some changes in outer membrane proteins, transcriptional regulators and T4SS proteins' expression, whereas ECH_0660 gene mutation had a minimal impact on the proteome.

## Author Contributions

RG conceived and directed the research design and CK designed research plan, executed and performed the experiments, and data analysis. HL contributed to *Ehrlichia* purification and 2D gel software analysis. CK and RG prepared the manuscript.

### Conflict of Interest Statement

The authors declare that the research was conducted in the absence of any commercial or financial relationships that could be construed as a potential conflict of interest.
