# Supplementary material for: Proteome Analysis Revealed Changes in Protein Expression Patterns Caused by Mutations in Ehrlichia chaffeensis
Source: Front Cell Infect Microbiol. 2019 Mar 18;9:58. doi: 10.3389/fcimb.2019.00058 (PMC6431617; doi:10.3389/fcimb.2019.00058)
Supplement: Supplementary file 2 [file Data_Sheet_1.PDF]

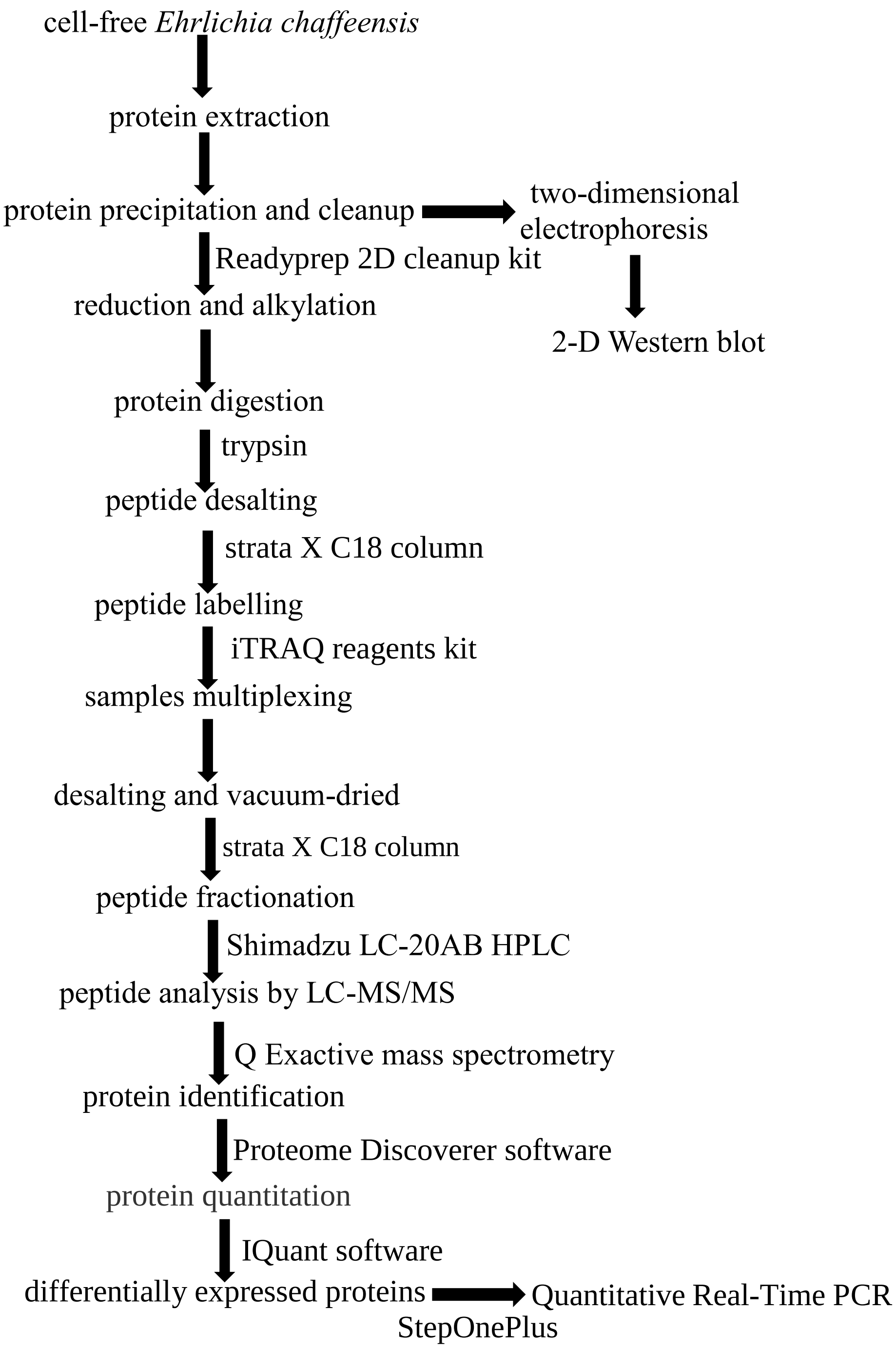

Supplementary Figure 1. Workflow of sample preparation, LC-MS/MS analysis and proteins quantitation of *E. chaffeensis* wildtype and mutants organisms.

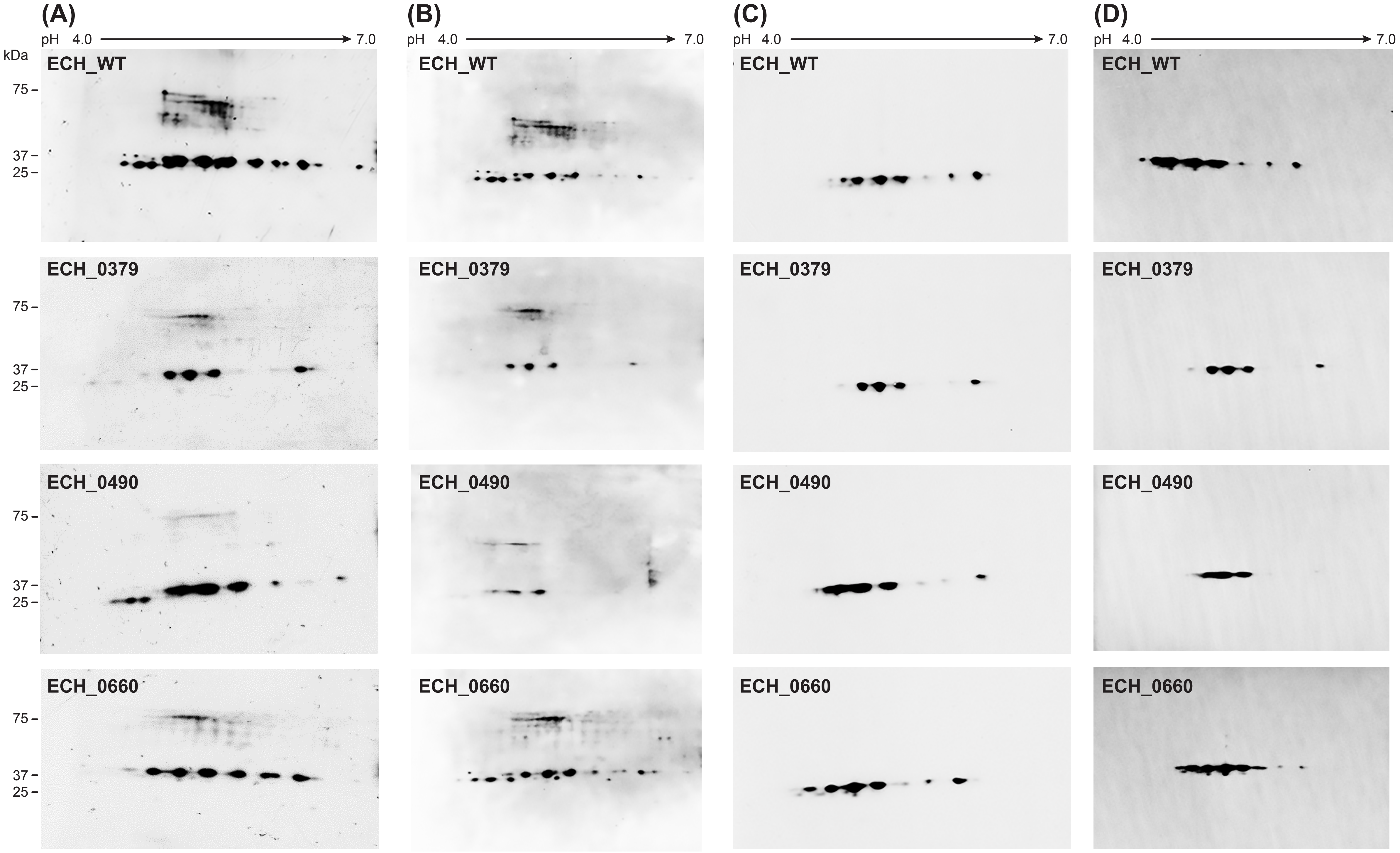

Supplementary Figure 2. Replicates of *E. chaffeensis* wildtype and mutants' proteomes immunolabelled with polyclonal sera (Panels A and B) and with p28 MAb (Panels C and D).

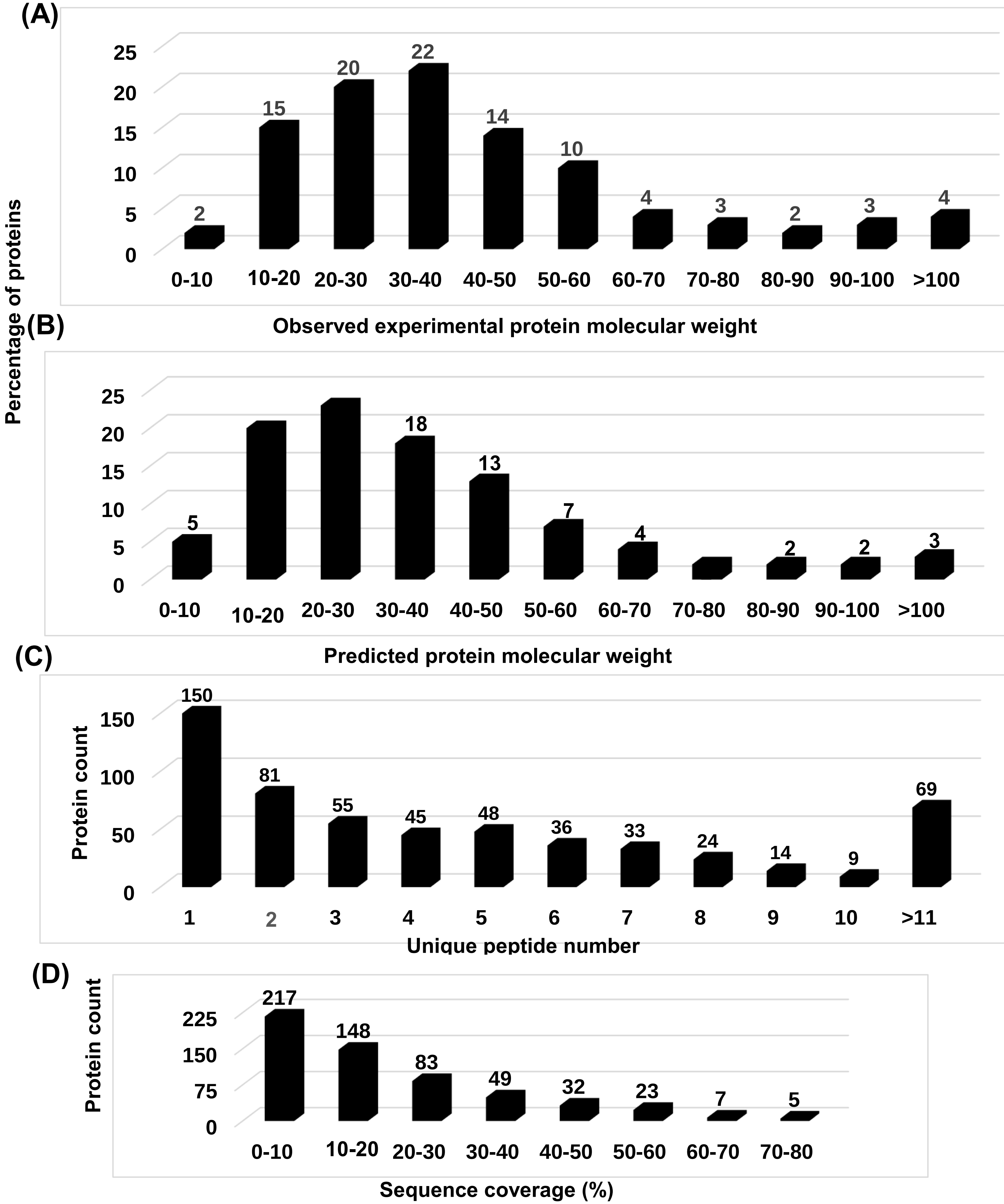

Supplementary Figure 3. Distribution of experimental (A) and predicted proteins molecular weights (B) obtained from proteomics data and genome sequence (NC_007799.1). Unique peptides of the detected proteins (C), and protein sequence coverage (D). The number of proteins in each of the categories is presented above each bar.
